# Deep venous thrombosis in ICU patients: exploring the submerged part of the iceberg by an expanded intra-ICU ultrasound surveillance program

**DOI:** 10.1186/cc9440

**Published:** 2011-03-11

**Authors:** A Cecchi, M Boddi, M Ciapetti, F Barbani, M Bonizzoli, J Parodo, L Perretta, G Zagli, E Spinelli, A Peris

**Affiliations:** 1 Careggi Teaching Hospital, Florence, Italy

## Introduction

Deep venous thrombosis (DVT) of lower extremities is a well-known complication in critically ill patients, but data for DVT prevalence in upper venous districts are rare. To explore the real prevalence of DVT in ICU patients, intensivists' routine ultrasound (US) surveillance was extended to include upper vein districts.

## Methods

This before-and-after intervention study included patients admitted to our ICU of a tertiary referral center for trauma and ECMO assistance (Careggi Teaching Hospital, Florence, Italy). The level I vascular US consists of evaluation of the lumen, and complete compressibility of the vein compression: it is performed by the intensivist on duty within the first 24 hours after ICU admission, every 7 days of the ICU stay or in cases of suspected DVT. A level II US examination is performed by a vascular specialist as a second opinion in cases of unclear or positive level I examinations. In 2010, the DVT surveillance protocol was extended to assess from lower extremities to include also the proximal upper extremities (axillary, brachial, cephalic veins) and internal jugular veins. DVTs already present at ICU admission were not included in the study, as well as central venous catheter (CVC)-related thrombosis less than 3 mm of thickness.

## Results

In 2009, 436 patients were admitted to our ICU (male sex 44%, mean age 57 years, mean SAPS II 36.6). Among the 436 patients admitted, a total of 466 level I examinations: eight cases of lower extremities DVT were diagnosed (1.8% of patients admitted) at level I examination. After introduction of expanding level I US surveillance (January to October 2010), 321 patients were admitted to our ICU (male sex 64%, mean age 55 years, mean SAPS II 37.6). A total of 358 level I examinations were performed. Expanding surveillance to upper venous districts, a significantly higher DVT rate (25 cases, 7.8%; *P *< 0.0001) at level I examination was found, all confirmed by the level II examination. In details, lower extremities DVTs were nine (2.8%), upper extremities DVTs 16 (5%), 11 of which were CVC-related at internal jugular vein. Mean time between admission and DVT diagnosis was 9.1 days.

## Conclusions

The lower extremities DVT represent only the tip of the DVT iceberg in critically ill patients. Our results suggest that routine intra-ICU US surveillance should include all venous districts, with particular care of those in which intravascular devices are positioned.

